# Crystal structure of bis­(pyridine-4-carbo­thio­amide-κ*N* ^1^)bis­(thio­cyanato-κ*N*)cobalt(II) methanol monosolvate

**DOI:** 10.1107/S2056989017015055

**Published:** 2017-10-31

**Authors:** Tristan Neumann, Inke Jess, Christian Näther

**Affiliations:** a Institut für Anorganische Chemie, Christian-Albrechts-Universität Kiel, Max-Eyth Strasse 2, D-24118 Kiel, Germany

**Keywords:** crystal structure, discrete complex, cobalt(II) thio­cyanate, pyridine-4-carbo­thio­amide, hydrogen bonding, methanol solvate

## Abstract

The asymmetric unit of the title compound, [Co(NCS)_2_(C_6_H_6_NS)_4_]·CH_3_OH, consists of one cobalt(II) cation, two thio­cyanate anions, four pyridine-4-carbo­thio­amide ligands and one methanol mol­ecule that are located in general positions. The Co^II^ cations are coordinated by two terminal N-bonding thio­cyanate anions and four N-bonding pyridine-4-carbo­thio­amide ligands, resulting in discrete and slightly distorted octa­hedral complexes. These complexes are linked into a three-dimensional network *via* inter­molecular N—H⋯S hydrogen bonding between the amino H atoms and the thio­cyanate and pyridine-4-carbo­thio­amide S atoms. From this arrangement, channels are formed in which the methanol solvate mol­ecules are embedded and linked to the host structure by inter­molecular O—H⋯S and N—H⋯O hydrogen bonding.

## Abstract 2

The crystal structure of the title compound consists of discrete octa­hedral complexes that are linked by inter­molecular N—H⋯S hydrogen bonds into a three-dimensional network that contains channels in which the hydrogen-bonded methanol mol­ecules are located.

## Chemical context   

Thio- and seleno­cyanate anions are useful ligands for the synthesis of new coordination compounds and polymers because of their versatile coordination behaviour (Massoud *et al.*, 2013 ▸; Kabesova *et al.*, 1995 ▸). Compounds in which the metal cations are linked by these ligands are of special inter­est because magnetic exchange can be mediated (Palion-Gazda *et al.*, 2015 ▸; Boeckmann & Näther, 2012 ▸; Wöhlert *et al.*, 2013 ▸). In this context, we are especially inter­ested in cobalt compounds in which the metal cations are octa­hedrally coordinated by two neutral co-ligands and four anionic ligands. In the corresponding structures, the central cations are linked into chains by mutual pairs of anionic ligands. Some of these compounds show a slow relaxation of the magnetization, which in part can be traced back to single-chain magnetism (Rams *et al.*, 2017*a* ▸,*b* ▸; Wöhlert *et al.*, 2012 ▸). To study the influence of the neutral co-ligand on the magnetic properties, different pyridine derivatives substituted in the 4-position, *e.g.* 4-benzoyl­pyridine, 4-vinyl­pyridine, 4-(4-chloro­benz­yl)pyridine and 4-(3-phenyl­prop­yl)pyridine have been investigated (Rams *et al.*, 2017*b* ▸; Werner *et al.*, 2014 ▸, 2015 ▸). In this regard, we also became inter­ested in pyridine-4-carbo­thio­amide as a ligand, because in this case the Co(NCS)_2_ chains can be linked into layers by pairs of inter­molecular hydrogen bonds between the amino H atoms and the thio­amide S atom. Unfortunately, the desired compound with composition Co(NCS)_2_(pyridine-4-carbo­thio­amide)_2_ could not be prepared from solution. Alternatively, we attempted to synthesize discrete solvato complexes as precursors that might transform into the desired compound on thermal annealing, as has been shown previously (Boeckmann & Näther, 2012 ▸). In the course of these investigations, crystals of the title compound were grown and characterized by single crystal X-ray diffraction. Unfortunately, no single-phase crystalline product could be obtained which prevented further investigations.
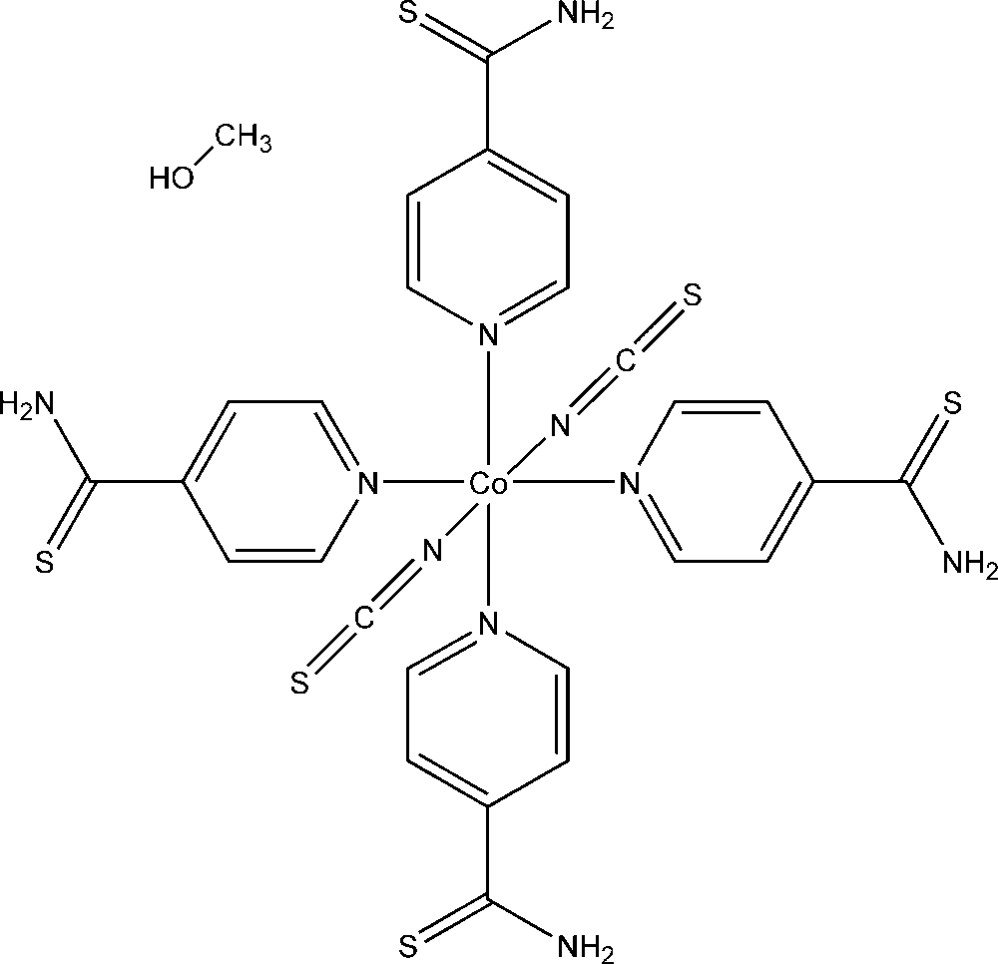


## Structural commentary   

**Figure 1:**
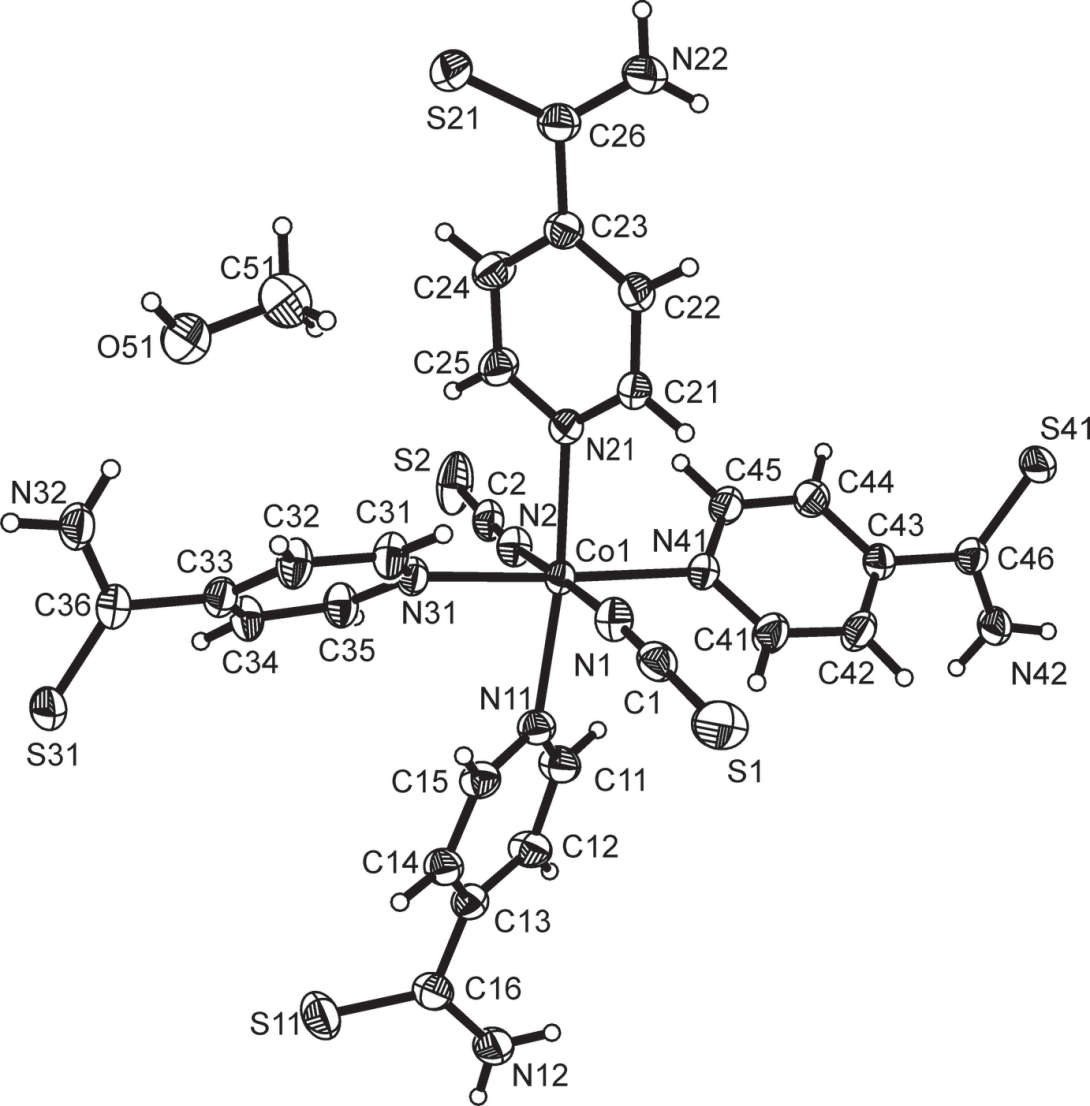
View of the asymmetric unit of the title compound, with atom labelling and displacement ellipsoids drawn at the 50% probability level.

The asymmetric unit of the title compound, [Co(NCS)_2_(C_6_H_6_NS)_4_]·CH_3_OH, consists of one Co^II^ cation, two thio­cyanate anions, four 4-pyridinde­thio­amide co-ligands and one one methanol mol­ecule, all located in general positions. The Co^II^ cation is sixfold coordinated by two terminal N-bonded thio­cyanate anions and four N-bonded pyridine-4-carbo­thio­amide ligands, resulting in discrete and slightly distorted octa­hedra (Fig. 1 ▸). The Co—N bond lengths to the thio­cyanate anions of 2.0847 (14) and 2.0865 (14) Å are significantly shorter then those to the pyridine N atoms of the pyridine-4-carbo­thio­amide ligand [2.1608 (13)–2.1933 (14) Å], in agreement with values reported in the literature (Goodgame *et al.*, 2003 ▸; Prananto *et al.*, 2017 ▸).

## Supra­molecular features   

**Figure 2:**
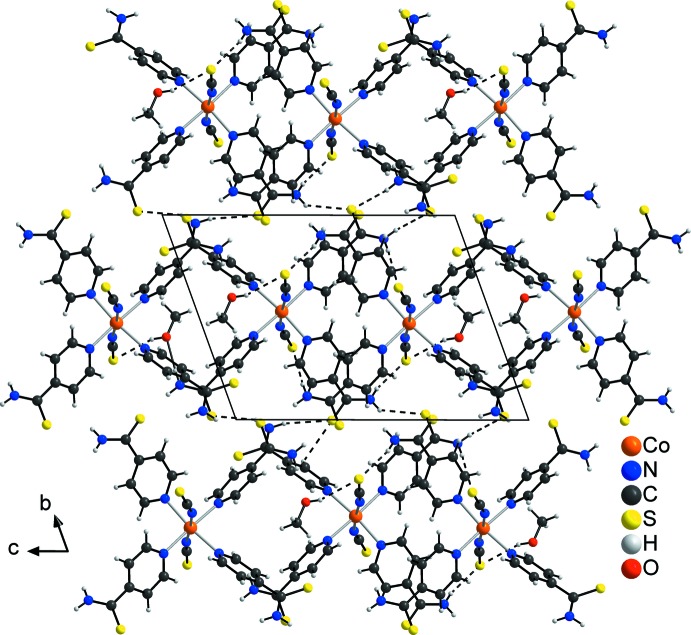
Crystal structure of the title compound in a view along the *a* axis. Inter­molecular hydrogen bonding is shown by dashed lines.

The discrete complexes are linked into a three-dimensional network by centrosymmetric pairs of inter­molecular N—H⋯S hydrogen bonds between the amino H atoms and the 4-pyridinde­thio­amide S atoms as well as by additional N—H⋯S hydrogen bonds involving the thio­cyanate S atoms (Fig. 2 ▸, Table 1 ▸). By this arrangement, channels extending parallel the *a* axis are formed in which the methanol solvate mol­ecules are located (Fig. 2 ▸). The solvent mol­ecules are connected to the network *via* inter­molecular O—H⋯S hydrogen bonding between the hydroxyl H atoms and the thio­cyanate S atoms (Table 1 ▸). It is noted that the methanol mol­ecules also act as acceptors for N—H⋯O hydrogen bonding from the amino group of neighbouring complexes. There are also additional short contacts between some of the aromatic hydrogen atoms and the two types of S atoms (thio­cyanate, 4-pyridinde­thio­amide), which are indicative of weak C—H⋯S inter­actions (Table 1 ▸).

## Database survey   

There are no structures of cobalt(II) thio­cyanate compounds with pyridine-4-carbo­thio­amide as co-ligand reported in the Cambridge Structure Database (Groom *et al.*, 2016 ▸). There is only one compound with cadmium, in which the Cd^II^ cations are octa­hedrally coordinated by two terminal N-bonded pyridine­thio­amide ligands and four thio­cyanate anions. The Cd^II^ cations are linked by pairs of the anionic ligands into linear chains, which corresponds exactly to the structure in which we were originally inter­ested (Neumann *et al.*, 2016 ▸).

## Synthesis and crystallization   

Co(NCS)_2_ and pyridine-4-carbo­thio­amide were purchased from Alfa Aesar. Crystals of the title compound suitable for single crystal X-ray diffraction were obtained by the reaction of 8.8 mg Co(NCS)_2_ (0.05 mmol) with 27.6 mg pyridine-4-carbo­thio­amide (0.2 mmol) in methanol (0.5 ml). The reaction mixture was heated to boiling and then left on the turned-off heating plate to cool down slowly. During this process, crystals of the title compound formed.

## Refinement   

Crystal data, data collection and structure refinement details are summarized in Table 2 ▸. The aromatic hydrogen atoms, the methyl hydrogen atoms and the hydrogen atom of the hy­droxy function were positioned with idealized geometry (the hy­droxy hydrogen atom was allowed to rotate but not to tip) and were refined with *U*_iso_(H) = 1.2*U*_eq_(C) (1.5 for methyl H atoms) using a riding model. The amino hydrogen atoms were located in a difference map. Their N—H bond lengths were set to ideal values and refined with *U*_iso_(H) = 1.5*U*_eq_(N).

## Supplementary Material

Crystal structure: contains datablock(s) I. DOI: 10.1107/S2056989017015055/wm5421sup1.cif

Structure factors: contains datablock(s) I. DOI: 10.1107/S2056989017015055/wm5421Isup2.hkl

CCDC reference: 1580309

Additional supporting information:  crystallographic information; 3D view; checkCIF report

## Figures and Tables

**Table 1:** Hydrogen-bond geometry (Å, °)

*D*—H⋯*A*	*D*—H	H⋯*A*	*D*⋯*A*	*D*—H⋯*A*
N12—H11*N*⋯S2^i^	0.88	2.45	3.3010 (16)	163
N12—H12*N*⋯S41^ii^	0.88	2.64	3.3589 (16)	140
C21—H21⋯S2^iii^	0.95	2.89	3.7626 (16)	153
N22—H21*N*⋯S31^iv^	0.88	2.69	3.4969 (17)	152
N22—H22*N*⋯S11^iv^	0.88	2.71	3.5691 (17)	166
C31—H31⋯N1	0.95	2.65	3.137 (2)	112
C34—H34⋯S21^v^	0.95	2.95	3.8809 (17)	165
C35—H35⋯S1^vi^	0.95	2.85	3.6906 (17)	148
C35—H35⋯N2	0.95	2.68	3.203 (2)	115
N32—H31*N*⋯O51	0.88	1.99	2.863 (2)	173
N32—H32*N*⋯S41^vii^	0.88	2.67	3.5176 (14)	163
N42—H41*N*⋯S1^viii^	0.88	2.49	3.3580 (16)	171
N42—H42*N*⋯S31^ix^	0.88	2.64	3.4935 (14)	165
O51—H51⋯S2^v^	0.84	2.43	3.1994 (17)	153

Symmetry codes: (i) 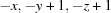; (ii) 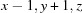; (iii) 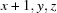; (iv) 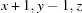; (v) 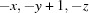; (vi) 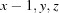; (vii) 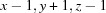; (viii) 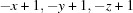; (ix) 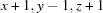.

**Table 2:** Experimental details

Crystal data	
Chemical formula	[Co(NCS)_2_(C_6_H_6_NS)_4_]·CH_4_O
*M*_r_	759.88
Crystal system, space group	Triclinic, *P* 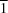
Temperature (K)	200
*a*, *b*, *c* (Å)	9.3136 (3), 12.4532 (5), 16.1799 (6)
α, β, γ (°)	70.584 (3), 89.453 (3), 74.996 (3)
*V* (Å^3^)	1703.51 (11)
*Z*	2
Radiation type	Mo *K*α
μ (mm^−1^)	0.91
Crystal size (mm)	0.15 × 0.10 × 0.06

Data collection	
Diffractometer	Stoe IPDS1
Absorption correction	Numerical (*X-RED32* and *X-SHAPE*; Stoe, 2008 ▸)
*T*_min_, *T*_max_	0.781, 0.926
No. of measured, independent and observed [*I* > 2σ(*I*)] reflections	26748, 8222, 6873
*R*_int_	0.029
(sin θ/λ)_max_ (Å^−1^)	0.661

Refinement	
*R*[*F* ^2^ > 2σ(*F* ^2^)], *wR*(*F* ^2^), *S*	0.031, 0.080, 1.07
No. of reflections	8222
No. of parameters	409
H-atom treatment	H-atom parameters constrained
Δρ_max_, Δρ_min_ (e Å^−3^)	0.34, −0.37

Computer programs: *X-AREA* (Stoe, 2008 ▸), *SHELXS97* (Sheldrick, 2008 ▸), *SHELXL2014* (Sheldrick, 2015 ▸), *publCIF* (Westrip, 2010 ▸) and *DIAMOND* (Brandenburg, 1999 ▸).

## supplementary crystallographic information

### Crystal data

**Table:**

[Co(NCS)_2_(C_6_H_6_NS)_4_]·CH_4_O	*Z* = 2
*M**_r_* = 759.88	*F*(000) = 782
Triclinic, *P*1	*D*_x_ = 1.481 Mg m^−^^3^
*a* = 9.3136 (3) Å	Mo *K*α radiation, λ = 0.71073 Å
*b* = 12.4532 (5) Å	Cell parameters from 26748 reflections
*c* = 16.1799 (6) Å	θ = 1.8–28.0°
α = 70.584 (3)°	µ = 0.91 mm^−^^1^
β = 89.453 (3)°	*T* = 200 K
γ = 74.996 (3)°	Block, violet
*V* = 1703.51 (11) Å^3^	0.15 × 0.10 × 0.06 mm

### Data collection

**Table:**

Stoe IPDS-1 diffractometer	6873 reflections with *I* > 2σ(*I*)
ω scans	*R*_int_ = 0.029
Absorption correction: numerical (X-RED32 and X-SHAPE; Stoe, 2008)	θ_max_ = 28.0°, θ_min_ = 1.8°
*T*_min_ = 0.781, *T*_max_ = 0.926	*h* = −12→12
26748 measured reflections	*k* = −16→16
8222 independent reflections	*l* = −21→21

### Refinement

**Table:**

Refinement on *F*^2^	Hydrogen site location: mixed
Least-squares matrix: full	H-atom parameters constrained
*R*[*F*^2^ > 2σ(*F*^2^)] = 0.031	*w* = 1/[σ^2^(*F*_o_^2^) + (0.0426*P*)^2^ + 0.3232*P*] where *P* = (*F*_o_^2^ + 2*F*_c_^2^)/3
*wR*(*F*^2^) = 0.080	(Δ/σ)_max_ = 0.001
*S* = 1.07	Δρ_max_ = 0.34 e Å^−^^3^
8222 reflections	Δρ_min_ = −0.37 e Å^−^^3^
409 parameters	Extinction correction: SHELXL2014 (Sheldrick, 2015), Fc^*^=kFc[1+0.001xFc^2^λ^3^/sin(2θ)]^-1/4^
0 restraints	Extinction coefficient: 0.0030 (7)

### Special details

**Table:**

Geometry. All esds (except the esd in the dihedral angle between two l.s. planes) are estimated using the full covariance matrix. The cell esds are taken into account individually in the estimation of esds in distances, angles and torsion angles; correlations between esds in cell parameters are only used when they are defined by crystal symmetry. An approximate (isotropic) treatment of cell esds is used for estimating esds involving l.s. planes.

### Fractional atomic coordinates and isotropic or equivalent isotropic displacement parameters (Å^2^)

**Table:**

	*x*	*y*	*z*	*U*_iso_*/*U*_eq_	
Co1	0.27982 (2)	0.47102 (2)	0.28964 (2)	0.02307 (7)	
N1	0.46073 (15)	0.54348 (13)	0.26565 (9)	0.0314 (3)	
C1	0.55593 (18)	0.58580 (14)	0.27195 (11)	0.0300 (3)	
S1	0.68741 (5)	0.64636 (5)	0.28192 (4)	0.04785 (13)	
N2	0.09728 (15)	0.40149 (12)	0.32138 (9)	0.0299 (3)	
C2	0.00111 (19)	0.35654 (15)	0.33143 (11)	0.0318 (3)	
S2	−0.13229 (6)	0.28999 (5)	0.34636 (4)	0.05500 (16)	
N11	0.17465 (15)	0.61256 (12)	0.34077 (9)	0.0281 (3)	
C11	0.1060 (2)	0.59454 (15)	0.41509 (11)	0.0334 (4)	
H11	0.1198	0.5155	0.4536	0.040*	
C12	0.0159 (2)	0.68505 (15)	0.43884 (11)	0.0344 (4)	
H12	−0.0278	0.6683	0.4934	0.041*	
C13	−0.00972 (19)	0.80101 (14)	0.38169 (11)	0.0294 (3)	
C14	0.06193 (19)	0.82030 (14)	0.30472 (11)	0.0307 (3)	
H14	0.0474	0.8982	0.2640	0.037*	
C15	0.15440 (19)	0.72529 (14)	0.28799 (11)	0.0292 (3)	
H15	0.2066	0.7402	0.2364	0.035*	
C16	−0.1113 (2)	0.90116 (15)	0.40209 (11)	0.0334 (4)	
S11	−0.24145 (7)	1.00294 (5)	0.32725 (3)	0.05019 (14)	
N12	−0.09385 (19)	0.90110 (14)	0.48257 (10)	0.0391 (3)	
H11N	−0.0195	0.8485	0.5191	0.059*	
H12N	−0.1533	0.9521	0.5031	0.059*	
N21	0.37050 (14)	0.34643 (12)	0.22359 (8)	0.0261 (3)	
C21	0.51800 (17)	0.30357 (14)	0.22423 (10)	0.0282 (3)	
H21	0.5816	0.3224	0.2597	0.034*	
C22	0.58254 (18)	0.23326 (14)	0.17595 (11)	0.0289 (3)	
H22	0.6880	0.2046	0.1787	0.035*	
C23	0.49188 (18)	0.20501 (14)	0.12339 (10)	0.0282 (3)	
C24	0.33894 (19)	0.24933 (16)	0.12248 (12)	0.0346 (4)	
H24	0.2730	0.2326	0.0870	0.041*	
C25	0.28281 (18)	0.31776 (16)	0.17335 (11)	0.0322 (3)	
H25	0.1777	0.3459	0.1729	0.039*	
C26	0.55584 (19)	0.13437 (15)	0.06639 (11)	0.0321 (3)	
S21	0.48865 (5)	0.17821 (4)	−0.03746 (3)	0.03921 (11)	
N22	0.6715 (2)	0.04167 (14)	0.10307 (11)	0.0461 (4)	
H21N	0.7089	0.0010	0.0689	0.069*	
H22N	0.7012	0.0187	0.1592	0.069*	
N31	0.16434 (14)	0.59080 (12)	0.16328 (8)	0.0258 (3)	
C31	0.24284 (18)	0.63003 (16)	0.09577 (10)	0.0313 (3)	
H31	0.3480	0.5977	0.1024	0.038*	
C32	0.17817 (18)	0.71544 (16)	0.01669 (11)	0.0327 (4)	
H32	0.2379	0.7391	−0.0303	0.039*	
C33	0.02558 (18)	0.76625 (14)	0.00659 (10)	0.0274 (3)	
C34	−0.05686 (18)	0.72709 (15)	0.07659 (11)	0.0310 (3)	
H34	−0.1616	0.7607	0.0726	0.037*	
C35	0.01598 (18)	0.63816 (15)	0.15246 (11)	0.0302 (3)	
H35	−0.0419	0.6092	0.1991	0.036*	
C36	−0.04773 (18)	0.85862 (15)	−0.07826 (10)	0.0288 (3)	
S31	−0.17011 (6)	0.98271 (4)	−0.07939 (3)	0.03822 (11)	
N32	−0.00718 (17)	0.83122 (13)	−0.14892 (9)	0.0343 (3)	
H31N	0.0501	0.7620	−0.1472	0.051*	
H32N	−0.0518	0.8772	−0.2010	0.051*	
N41	0.39825 (15)	0.34459 (12)	0.41333 (9)	0.0274 (3)	
C41	0.4822 (2)	0.37337 (15)	0.46472 (11)	0.0361 (4)	
H41	0.4923	0.4517	0.4457	0.043*	
C42	0.5548 (2)	0.29474 (15)	0.54422 (11)	0.0367 (4)	
H42	0.6105	0.3199	0.5795	0.044*	
C43	0.54595 (18)	0.17943 (14)	0.57193 (10)	0.0278 (3)	
C44	0.4615 (2)	0.14806 (14)	0.51807 (11)	0.0330 (4)	
H44	0.4534	0.0693	0.5343	0.040*	
C45	0.3894 (2)	0.23294 (14)	0.44069 (11)	0.0323 (3)	
H45	0.3304	0.2108	0.4050	0.039*	
C46	0.62759 (18)	0.09095 (14)	0.65609 (10)	0.0285 (3)	
S41	0.76058 (5)	−0.02709 (4)	0.65401 (3)	0.03816 (11)	
N42	0.59116 (17)	0.11527 (13)	0.72785 (9)	0.0342 (3)	
H41N	0.5124	0.1720	0.7285	0.051*	
H42N	0.6389	0.0731	0.7799	0.051*	
O51	0.1577 (2)	0.59710 (15)	−0.13645 (10)	0.0604 (4)	
H51	0.1793	0.6081	−0.1886	0.091*	
C51	0.2618 (3)	0.4961 (2)	−0.07977 (18)	0.0658 (7)	
H51A	0.3545	0.5159	−0.0713	0.099*	
H51B	0.2823	0.4334	−0.1057	0.099*	
H51C	0.2209	0.4686	−0.0229	0.099*	

### Atomic displacement parameters (Å^2^)

**Table:**

	*U*^11^	*U*^22^	*U*^33^	*U*^12^	*U*^13^	*U*^23^
Co1	0.02305 (11)	0.02408 (11)	0.01983 (11)	−0.00347 (8)	−0.00100 (7)	−0.00676 (8)
N1	0.0281 (7)	0.0329 (7)	0.0307 (7)	−0.0080 (6)	−0.0002 (5)	−0.0080 (6)
C1	0.0281 (8)	0.0293 (8)	0.0294 (8)	−0.0015 (6)	0.0040 (6)	−0.0109 (6)
S1	0.0289 (2)	0.0497 (3)	0.0759 (4)	−0.0142 (2)	0.0085 (2)	−0.0329 (3)
N2	0.0279 (7)	0.0324 (7)	0.0271 (7)	−0.0077 (6)	0.0011 (5)	−0.0073 (5)
C2	0.0301 (8)	0.0346 (8)	0.0236 (7)	−0.0040 (7)	−0.0031 (6)	−0.0044 (6)
S2	0.0428 (3)	0.0648 (3)	0.0469 (3)	−0.0303 (3)	−0.0122 (2)	0.0069 (2)
N11	0.0322 (7)	0.0265 (6)	0.0260 (6)	−0.0070 (5)	0.0032 (5)	−0.0102 (5)
C11	0.0453 (10)	0.0263 (8)	0.0259 (8)	−0.0083 (7)	0.0061 (7)	−0.0067 (6)
C12	0.0477 (10)	0.0293 (8)	0.0254 (8)	−0.0087 (7)	0.0099 (7)	−0.0097 (6)
C13	0.0341 (8)	0.0284 (8)	0.0257 (7)	−0.0074 (6)	0.0022 (6)	−0.0100 (6)
C14	0.0393 (9)	0.0252 (7)	0.0260 (8)	−0.0083 (7)	0.0026 (7)	−0.0071 (6)
C15	0.0354 (8)	0.0291 (8)	0.0248 (7)	−0.0108 (6)	0.0058 (6)	−0.0100 (6)
C16	0.0409 (9)	0.0284 (8)	0.0288 (8)	−0.0069 (7)	0.0061 (7)	−0.0093 (6)
S11	0.0599 (3)	0.0417 (3)	0.0312 (2)	0.0116 (2)	−0.0026 (2)	−0.00872 (19)
N12	0.0483 (9)	0.0359 (8)	0.0302 (7)	−0.0006 (7)	0.0017 (7)	−0.0158 (6)
N21	0.0254 (6)	0.0280 (6)	0.0242 (6)	−0.0041 (5)	0.0003 (5)	−0.0103 (5)
C21	0.0250 (7)	0.0312 (8)	0.0271 (8)	−0.0032 (6)	−0.0024 (6)	−0.0116 (6)
C22	0.0257 (7)	0.0297 (8)	0.0281 (8)	−0.0022 (6)	0.0002 (6)	−0.0099 (6)
C23	0.0318 (8)	0.0264 (7)	0.0255 (7)	−0.0068 (6)	0.0036 (6)	−0.0085 (6)
C24	0.0308 (8)	0.0432 (9)	0.0378 (9)	−0.0120 (7)	0.0035 (7)	−0.0226 (8)
C25	0.0249 (7)	0.0411 (9)	0.0354 (9)	−0.0079 (7)	0.0020 (6)	−0.0200 (7)
C26	0.0341 (8)	0.0313 (8)	0.0321 (8)	−0.0071 (7)	0.0046 (7)	−0.0136 (7)
S21	0.0365 (2)	0.0477 (3)	0.0331 (2)	−0.00146 (19)	−0.00106 (18)	−0.02079 (19)
N22	0.0574 (10)	0.0387 (8)	0.0337 (8)	0.0081 (7)	−0.0027 (7)	−0.0175 (7)
N31	0.0243 (6)	0.0291 (6)	0.0203 (6)	−0.0036 (5)	−0.0007 (5)	−0.0067 (5)
C31	0.0235 (7)	0.0419 (9)	0.0239 (8)	−0.0050 (7)	0.0009 (6)	−0.0081 (7)
C32	0.0262 (8)	0.0435 (9)	0.0224 (7)	−0.0080 (7)	0.0017 (6)	−0.0046 (7)
C33	0.0285 (7)	0.0288 (7)	0.0223 (7)	−0.0065 (6)	−0.0020 (6)	−0.0063 (6)
C34	0.0233 (7)	0.0350 (8)	0.0277 (8)	−0.0030 (6)	−0.0010 (6)	−0.0054 (6)
C35	0.0251 (7)	0.0357 (8)	0.0242 (8)	−0.0065 (6)	0.0018 (6)	−0.0042 (6)
C36	0.0272 (7)	0.0319 (8)	0.0238 (7)	−0.0081 (6)	−0.0019 (6)	−0.0047 (6)
S31	0.0474 (3)	0.0316 (2)	0.0262 (2)	0.00146 (18)	−0.00544 (18)	−0.00684 (16)
N32	0.0354 (7)	0.0369 (8)	0.0229 (7)	−0.0026 (6)	−0.0028 (6)	−0.0055 (6)
N41	0.0302 (7)	0.0261 (6)	0.0225 (6)	−0.0041 (5)	−0.0043 (5)	−0.0067 (5)
C41	0.0490 (10)	0.0265 (8)	0.0295 (8)	−0.0110 (7)	−0.0111 (7)	−0.0044 (6)
C42	0.0491 (10)	0.0305 (8)	0.0287 (8)	−0.0116 (7)	−0.0134 (7)	−0.0066 (7)
C43	0.0288 (7)	0.0268 (7)	0.0239 (7)	−0.0023 (6)	−0.0022 (6)	−0.0072 (6)
C44	0.0400 (9)	0.0251 (8)	0.0316 (8)	−0.0085 (7)	−0.0052 (7)	−0.0067 (6)
C45	0.0374 (9)	0.0283 (8)	0.0294 (8)	−0.0085 (7)	−0.0071 (7)	−0.0076 (6)
C46	0.0312 (8)	0.0272 (7)	0.0245 (7)	−0.0063 (6)	−0.0026 (6)	−0.0065 (6)
S41	0.0462 (3)	0.0309 (2)	0.0285 (2)	0.00573 (18)	−0.00729 (18)	−0.01084 (16)
N42	0.0372 (8)	0.0336 (7)	0.0243 (7)	0.0010 (6)	−0.0035 (6)	−0.0081 (6)
O51	0.0771 (11)	0.0529 (9)	0.0403 (8)	0.0034 (8)	−0.0046 (8)	−0.0176 (7)
C51	0.0840 (18)	0.0442 (12)	0.0592 (15)	−0.0041 (12)	−0.0121 (13)	−0.0144 (11)

### Geometric parameters (Å, º)

**Table:**

Co1—N1	2.0847 (14)	N22—H21N	0.8800
Co1—N2	2.0865 (14)	N22—H22N	0.8800
Co1—N21	2.1608 (13)	N31—C31	1.335 (2)
Co1—N31	2.1783 (12)	N31—C35	1.342 (2)
Co1—N41	2.1792 (13)	C31—C32	1.382 (2)
Co1—N11	2.1933 (14)	C31—H31	0.9500
N1—C1	1.164 (2)	C32—C33	1.384 (2)
C1—S1	1.6300 (18)	C32—H32	0.9500
N2—C2	1.157 (2)	C33—C34	1.386 (2)
C2—S2	1.6381 (18)	C33—C36	1.495 (2)
N11—C11	1.336 (2)	C34—C35	1.384 (2)
N11—C15	1.343 (2)	C34—H34	0.9500
C11—C12	1.384 (2)	C35—H35	0.9500
C11—H11	0.9500	C36—N32	1.323 (2)
C12—C13	1.391 (2)	C36—S31	1.6605 (17)
C12—H12	0.9500	N32—H31N	0.8800
C13—C14	1.386 (2)	N32—H32N	0.8799
C13—C16	1.489 (2)	N41—C45	1.336 (2)
C14—C15	1.377 (2)	N41—C41	1.336 (2)
C14—H14	0.9500	C41—C42	1.381 (2)
C15—H15	0.9500	C41—H41	0.9500
C16—N12	1.314 (2)	C42—C43	1.379 (2)
C16—S11	1.6594 (18)	C42—H42	0.9500
N12—H11N	0.8799	C43—C44	1.389 (2)
N12—H12N	0.8800	C43—C46	1.495 (2)
N21—C21	1.338 (2)	C44—C45	1.380 (2)
N21—C25	1.346 (2)	C44—H44	0.9500
C21—C22	1.383 (2)	C45—H45	0.9500
C21—H21	0.9500	C46—N42	1.314 (2)
C22—C23	1.388 (2)	C46—S41	1.6690 (17)
C22—H22	0.9500	N42—H41N	0.8800
C23—C24	1.386 (2)	N42—H42N	0.8800
C23—C26	1.496 (2)	O51—C51	1.408 (3)
C24—C25	1.380 (2)	O51—H51	0.8400
C24—H24	0.9500	C51—H51A	0.9800
C25—H25	0.9500	C51—H51B	0.9800
C26—N22	1.326 (2)	C51—H51C	0.9800
C26—S21	1.6572 (18)		
			
N1—Co1—N2	176.11 (6)	N22—C26—C23	115.73 (15)
N1—Co1—N21	92.32 (5)	N22—C26—S21	124.05 (14)
N2—Co1—N21	91.06 (5)	C23—C26—S21	120.11 (12)
N1—Co1—N31	91.36 (5)	C26—N22—H21N	115.1
N2—Co1—N31	90.73 (5)	C26—N22—H22N	121.5
N21—Co1—N31	87.09 (5)	H21N—N22—H22N	123.0
N1—Co1—N41	89.20 (5)	C31—N31—C35	117.36 (13)
N2—Co1—N41	88.87 (5)	C31—N31—Co1	119.81 (10)
N21—Co1—N41	90.24 (5)	C35—N31—Co1	122.41 (11)
N31—Co1—N41	177.29 (5)	N31—C31—C32	123.04 (15)
N1—Co1—N11	87.53 (6)	N31—C31—H31	118.5
N2—Co1—N11	89.35 (5)	C32—C31—H31	118.5
N21—Co1—N11	172.94 (5)	C31—C32—C33	119.40 (16)
N31—Co1—N11	85.85 (5)	C31—C32—H32	120.3
N41—Co1—N11	96.82 (5)	C33—C32—H32	120.3
C1—N1—Co1	163.69 (14)	C32—C33—C34	118.04 (14)
N1—C1—S1	178.93 (16)	C32—C33—C36	120.67 (15)
C2—N2—Co1	171.72 (14)	C34—C33—C36	121.28 (14)
N2—C2—S2	178.71 (16)	C35—C34—C33	118.87 (15)
C11—N11—C15	116.99 (14)	C35—C34—H34	120.6
C11—N11—Co1	123.49 (11)	C33—C34—H34	120.6
C15—N11—Co1	118.44 (11)	N31—C35—C34	123.21 (15)
N11—C11—C12	123.40 (15)	N31—C35—H35	118.4
N11—C11—H11	118.3	C34—C35—H35	118.4
C12—C11—H11	118.3	N32—C36—C33	114.27 (15)
C11—C12—C13	119.03 (16)	N32—C36—S31	124.92 (12)
C11—C12—H12	120.5	C33—C36—S31	120.80 (12)
C13—C12—H12	120.5	C36—N32—H31N	124.0
C14—C13—C12	117.78 (15)	C36—N32—H32N	120.1
C14—C13—C16	121.00 (15)	H31N—N32—H32N	114.8
C12—C13—C16	121.22 (15)	C45—N41—C41	117.06 (14)
C15—C14—C13	119.26 (15)	C45—N41—Co1	120.25 (10)
C15—C14—H14	120.4	C41—N41—Co1	122.68 (11)
C13—C14—H14	120.4	N41—C41—C42	123.16 (16)
N11—C15—C14	123.42 (16)	N41—C41—H41	118.4
N11—C15—H15	118.3	C42—C41—H41	118.4
C14—C15—H15	118.3	C43—C42—C41	119.50 (15)
N12—C16—C13	115.95 (15)	C43—C42—H42	120.2
N12—C16—S11	123.07 (14)	C41—C42—H42	120.2
C13—C16—S11	120.95 (13)	C42—C43—C44	117.72 (14)
C16—N12—H11N	120.2	C42—C43—C46	120.89 (14)
C16—N12—H12N	123.4	C44—C43—C46	121.37 (14)
H11N—N12—H12N	116.3	C45—C44—C43	119.03 (15)
C21—N21—C25	117.15 (14)	C45—C44—H44	120.5
C21—N21—Co1	120.78 (10)	C43—C44—H44	120.5
C25—N21—Co1	121.75 (11)	N41—C45—C44	123.49 (15)
N21—C21—C22	123.32 (14)	N41—C45—H45	118.3
N21—C21—H21	118.3	C44—C45—H45	118.3
C22—C21—H21	118.3	N42—C46—C43	116.01 (14)
C21—C22—C23	119.39 (15)	N42—C46—S41	124.26 (12)
C21—C22—H22	120.3	C43—C46—S41	119.69 (12)
C23—C22—H22	120.3	C46—N42—H41N	122.5
C24—C23—C22	117.47 (15)	C46—N42—H42N	123.2
C24—C23—C26	120.86 (15)	H41N—N42—H42N	114.2
C22—C23—C26	121.61 (15)	C51—O51—H51	109.5
C25—C24—C23	119.77 (15)	O51—C51—H51A	109.5
C25—C24—H24	120.1	O51—C51—H51B	109.5
C23—C24—H24	120.1	H51A—C51—H51B	109.5
N21—C25—C24	122.88 (15)	O51—C51—H51C	109.5
N21—C25—H25	118.6	H51A—C51—H51C	109.5
C24—C25—H25	118.6	H51B—C51—H51C	109.5

### Hydrogen-bond geometry (Å, º)

**Table:**

*D*—H···*A*	*D*—H	H···*A*	*D*···*A*	*D*—H···*A*
N12—H11*N*···S2^i^	0.88	2.45	3.3010 (16)	163
N12—H12*N*···S41^ii^	0.88	2.64	3.3589 (16)	140
C21—H21···S2^iii^	0.95	2.89	3.7626 (16)	153
N22—H21*N*···S31^iv^	0.88	2.69	3.4969 (17)	152
N22—H22*N*···S11^iv^	0.88	2.71	3.5691 (17)	166
C31—H31···N1	0.95	2.65	3.137 (2)	112
C34—H34···S21^v^	0.95	2.95	3.8809 (17)	165
C35—H35···S1^vi^	0.95	2.85	3.6906 (17)	148
C35—H35···N2	0.95	2.68	3.203 (2)	115
N32—H31*N*···O51	0.88	1.99	2.863 (2)	173
N32—H32*N*···S41^vii^	0.88	2.67	3.5176 (14)	163
N42—H41*N*···S1^viii^	0.88	2.49	3.3580 (16)	171
N42—H42*N*···S31^ix^	0.88	2.64	3.4935 (14)	165
O51—H51···S2^v^	0.84	2.43	3.1994 (17)	153

Symmetry codes: (i) −*x*, −*y*+1, −*z*+1; (ii) *x*−1, *y*+1, *z*; (iii) *x*+1, *y*, *z*; (iv) *x*+1, *y*−1, *z*; (v) −*x*, −*y*+1, −*z*; (vi) *x*−1, *y*, *z*; (vii) *x*−1, *y*+1, *z*−1; (viii) −*x*+1, −*y*+1, −*z*+1; (ix) *x*+1, *y*−1, *z*+1.
